# Adaptor protein CIN85 potentiates the motility of osteosarcoma cells via the Akt/mTOR and MMP2‐COL3A1 axis

**DOI:** 10.1002/1878-0261.70245

**Published:** 2026-04-17

**Authors:** Iryna Horak, Iva Staniczková Zambo, Matěj Přikryl, Peter Múdry, Danica Zapletalová, Jarmila Navrátilová, Jiří Navrátil, Jan Šmarda, Liudmyla Drobot, Petr Beneš, Lucia Knopfová

**Affiliations:** ^1^ Faculty of Science, Department of Experimental Biology Masaryk University Brno Czech Republic; ^2^ Department of Cell Signaling Palladin Institute of Biochemistry, National Academy of Sciences of Ukraine Kyiv Ukraine; ^3^ International Clinical Research Center, St. Anne's University Hospital Brno Czech Republic; ^4^ 1st Department of Pathology, St. Anne's University Hospital and Faculty of Medicine Masaryk University Brno Czech Republic; ^5^ Department of Pediatric Oncology, University Hospital Brno and Faculty of Medicine Masaryk University Brno Czech Republic; ^6^ Department of Pathological Physiology, Faculty of Medicine Masaryk University Brno Czech Republic

**Keywords:** CIN85, metastasis, migration, osteosarcoma

## Abstract

Osteosarcoma is the most common malignant bone tumor, primarily affecting adolescents and young adults. Patients with metastases have a low survival rate, making the identification of prognostic markers crucial. The adaptor protein CIN85 is involved in various signaling pathways that regulate cell differentiation, adhesion, and motility. Its overexpression is associated with poor prognosis in multiple cancers. However, the role of CIN85 in osteosarcoma progression has not yet been explored. This study shows that CIN85 expression is higher in osteosarcoma than in normal bone tissue and further increased in metastatic lesions relative to primary tumors. CIN85 overexpression increases cell migration and Matrigel invasion, whereas silencing CIN85 suppresses these behaviors. Functional annotation and enrichment analyses of the CIN85‐driven transcriptome suggest that CIN85 regulates migration, adhesion, and extracellular matrix organization in osteosarcoma. CIN85 affects *MMP2* and *COL3A1* gene expression and activates Akt/mTOR signaling. Knockdown of *MMP2* and *COL3A1* or pharmacological inhibion of Akt/mTOR signaling abrogates CIN85‐induced motility. This study demonstrates that elevated CIN85 expression contributes to osteosarcoma migration and metastasis, highlighting its potential as a therapeutic target.

## Graphical abstract

CIN85 is highly expressed in osteosarcoma, particularly in metastatic lesions. Its overexpression increases cell migration and Matrigel invasion, while silencing CIN85 suppresses these behaviors. Transcriptome analysis shows that CIN85 regulates *MMP2, COL3A1*, and Akt/mTOR signaling. Targeting these pathways reverses CIN85‐induced motility, highlighting CIN85 as a potential therapeutic target.

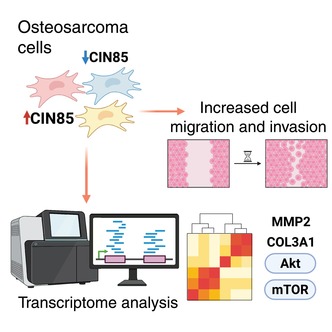

**Abbreviations**
Akt: protein kinase B
CIN85: Cbl‐interacting protein of 85 kDa
COL3A1: collagen type III alpha 1 chain
DEGs: differentially expressed genes
HCLS1: hematopoietic cell‐specific lyn substrate 1
IHC: immunohistochemistry
MMP2: matrix metallopeptidase 2
MMPs: matrix metalloproteinases
mTOR: mechanistic target of rapamycin kinase
OSA: osteosarcoma
qPCR: quantitative polymerase chain reaction
SH3KBP1: SH3 domain containing kinase binding protein 1
siRNA: small interfering RNA

## 1 Introduction

Osteosarcoma (OSA) is the most common primary malignant bone cancer mainly affecting children and young adults that accounts for approximately 8.9% of cancer‐related deaths in children. OSA most frequently occurs in the femur, tibia, and humerus, rarely in the skull, mandible, pelvis, and ribs. OSA metastasizes mainly to the lungs and other bones; about 20% of patients have metastases at diagnosis, and the 5‐year survival rate is about 30%. Treatment options include surgery, standard chemotherapy with methotrexate, doxorubicin, cisplatin, as well as immunotherapy [1, 2, 3, 4, 5, 6]. Despite considerable progress in OSA treatment, the use and effectiveness of targeted therapy remain limited. Consequently, identifying and validating reliable prognostic markers to detect patients at risk and guiding personalized therapy has become an urgent priority.

Genomic advances have identified genetic alterations and dysregulated signaling cascades associated with high‐grade OSA. For example, OSA patients often have p53 mutations and elevated IGFR, PI3K, and MAPK signaling [5]. In addition, OSA cells undergo metabolic reprogramming driven by PI3K/Akt/mTOR, HIF‐1, Wnt, Hippo, NF‐κB, and MAPK signaling [2]. Recent clinical trials have investigated PI3K/Akt/mTOR inhibitors, tyrosine kinase inhibitors, and angiogenesis modulators, but have achieved only partial responses and/or transient disease stabilization [7]. The overall efficacy of targeted therapies is limited due to tumor heterogeneity, lack of predictive biomarkers, genetic instability, and rapid emergence of drug resistance [7, 8]. To overcome these limitations, a shift toward precision bone oncology endowed with predictive biomarkers guiding patient selection is critically needed.

The adaptor protein CIN85, encoded by the *SH3KBP1* gene, is a member of the CIN85/CMS family of adaptor molecules [9]. CIN85 has been reported to be overexpressed in various cancers, including breast [10, 11], prostate [12], and cervical carcinoma [13]. In addition, its increased expression is associated with an unfavorable prognosis in breast carcinoma [10, 11], prostate cancer [12], esophageal squamous cell carcinoma [14], cervical cancer [13], colon cancer [15], and glioblastoma [16]. CIN85 interacts with various proteins and is thereby involved in vesicle‐mediated transport, cell adhesion, migration, and invasiveness [9, 17].

CIN85 plays a significant role in the progression of various cancers and may serve as a prognostic marker. Recently, the *SH3KBP1* gene was identified as one of the upregulated genes in metastatic OSA cell lines [18]. However, there is a lack of information regarding its functional involvement in tumorigenesis and dissemination of non‐epithelial tumors, including osteosarcoma. Therefore, this study aimed to evaluate CIN85 expression in OSA and to investigate its role in the biological processes and signaling involved in the progression of the OSA tumors.

## 2 Materials and methods

### 2.1 Data mining

The TNMplot database (tnmplot.com) [19] was used to access *SH3KBP1* expression data obtained by RNA‐seq in human osteosarcoma samples (OS‐TARGET dataset) compared to normal samples from non‐cancerous patients and additional pediatric tissues. Publicly available NCBI GEO (Gene Expression Omnibus) datasets GSE66674, GSE119975, GSE49003, GSE220538, GSE237033, GSE87624, and GSE32981 were analyzed to compare *SH3KBP1* expression between parental and metastatic cell lines, as well as between primary tumors and metastases. Expression values obtained from each GEO dataset represent the TPM normalized counts (for RNA‐seq) or the “Value” column of the original submitter‐supplied Sample record (for microarrays). For the RNA‐seq datasets GSE220538, GSE237033, GSE87624, and GSE32981, normalization was performed independently by calculating the median expression value within each dataset and scaling all expression measurements by median value. The normalized datasets were then merged into a single dataset for downstream analyses. Statistical comparisons were conducted using Student's *t*‐test with Welch's correction and, additionally, the non‐parametric Mann–Whitney test. Kaplan–Meier plots for metastasis‐free survival of osteosarcoma patients (dataset GSE42352) were retrieved from the R2 Genomics Analysis and Visualization Platform (https://hgserver1.amc.nl/cgi‐bin/r2/main.cgi?option=kaplan_main).

### 2.2 Patients

The study group consisted of 20 pediatric patients [12 males and 8 females; median age 14 years (range 3–17 years)] with high‐grade OSA who underwent treatment in the Department of Pediatric Oncology, University Hospital Brno. All cases were diagnosed according to the criteria specified in the current WHO Classification of Tumors of Soft Tissue and Bone [20]. Samples were collected during the period 2007–2020 at the Department of Pediatric Oncology, University Hospital Brno. Written informed consent was obtained from patients or their legal guardians, and the study was approved by the Ethics Committee of the University Hospital Brno (13‐120 423/EK) and Masaryk University (EKV‐2023‐064). All procedures were conducted in accordance with the Declaration of Helsinki. Only samples that did not require decalcification were included, as this procedure may alter protein detection and cause false‐negative results in immunohistochemical (IHC) detection [21]. Tissue specimens were fixed in 10% neutral buffered formalin and embedded in paraffin. Tissue sections were stained with hematoxylin–eosin and examined by pathologists specializing in sarcomas. Representative tissue blocks were selected for IHC analysis. All tumor tissue samples were obtained prior to chemotherapy or radiotherapy.

### 2.3 Immunohistochemistry

Formalin‐fixed paraffin‐embedded tissue block samples were sectioned at a 2 μm thickness using a microtome (Leica SM 2010R) and mounted on positively charged slides. IHC was performed using an automated immunostainer (BenchMark ULTRA, Ventana Medical Systems, Tucson, Arizona, USA). Antigen retrieval was performed with CC1 antigen retrieval solution (prediluted; pH 8.0, ref. 950–124, Ventana Medical Systems) for 36 min at 95 °C. Specimens were incubated with a primary antibody directed against CIN85 (PA5‐51903, Thermo Fisher Scientific, Waltham, MA) at a concentration of 1 : 1000 for 32 min at 36 °C, followed by visualization with the Ultra View DAB IHC Detection Kit (Ventana Medical Systems). Slides were counterstained with hematoxylin Gill for 1 min (Merck, Darmstadt, Germany), blued in tap water, and coverslipped. Each IHC run included a positive control (on‐slide tonsil) and a negative antibody control (buffer without primary antibody).

### 2.4 Cell culture

Osteosarcoma cell lines HOS (RRID:CVCL_0312) and SAOS‐2 (RRID:CVCL_0548) were kindly provided by Prof. Bruno Fuchs [22]. All cell lines were authenticated by Generi Biotech using short tandem repeat profiling. Cells were cultured at 37 °C in a humidified atmosphere with 5% CO_2_ in complete DMEM (Sigma‐Aldrich, St. Louis, MO) supplemented with 10% fetal bovine serum (FBS) (Invitrogen, Carlsbad, CA), 100 U·mL^−1^ penicillin and 100 μg·mL^−1^ streptomycin (Lonza, Basel), and 2 mm L‐glutamine. All cell lines were regularly tested for mycoplasma contamination.

To generate upCIN85 cells, parental HOS and SAOS‐2 cells were transfected with the pRc/CMV2‐Ruk_l_ plasmid [11] encoding the full‐length form of CIN85 or with an empty vector (mock) using Lipofectamine 3000 (Invitrogen, Carlsbad, CA). Cells were selected with 2 mg·mL^−1^ geneticin (G418) (Sigma‐Aldrich, St. Louis, MO). Geneticin‐resistant pool was analyzed for CIN85 expression by western blotting and used in further experiments.

For gene silencing, commercially available siRNAs were used: a mix of three CIN85‐targeting siRNAs (SR309344; OriGene Technologies, Rockville, MD), *HCLS1* siRNA (s6482; ThermoFisher Scientific), *MMP2* siRNAs (s8851 and s8852; ThermoFisher Scientific), and *COL3A1* siRNAs (s3284 and s3285; ThermoFisher Scientific).

For pathway inhibitor studies, cells were treated with 1 μm MK‐2206 (11 593; Cayman Chemical, Michigan, USA; Akt inhibitor) and 0.5 μm rapamycin (553 210; Sigma‐Aldrich, St. Louis, MO; mTOR inhibitor) for 24 h.

### 2.5 Western blotting

Cells were scraped into the lysis buffer and lysed as described previously [23]. Proteins were separated by SDS/PAGE and transferred to PVDF membranes. Blots were incubated with primary antibodies against CIN85 (PA5‐51903; ThermoFisher Scientific, Waltham, MA), p‐Akt Ser473 (4060; Cell Signaling Technology), Akt (9272; Cell Signaling Technology), p‐mTOR Ser2448 (5536; Cell Signaling Technology), mTOR (2972; Cell Signaling Technology), p‐SAPK/JNK Thr183/Tyr185 (9251; Cell Signaling Technology), pERK1/2 T202/Y204 (ab214362; Abcam), p‐p38 T180/Y182 (9216; Cell Signaling Technology), pIκBα S32 (2859; Cell Signaling Technology), pEGFR Y1068 (3777; Cell Signaling Technology), EGFR (4267; Cell Signaling Technology), MMP‐2 (4022; Cell Signaling Technology), COL3A1 (30565; Cell Signaling Technology), fibronectin (bd610077; BD Biosciences), N‐cadherin (ab765011; Abcam), vimentin (5741; Cell Signaling Technology), SNAIL/SLUG (ab180714; Abcam), TGFβ (3711; Cell Signaling Technology), or α‐tubulin (T9026; Sigma‐Aldrich), followed by HRP‐conjugated mouse or rabbit secondary antibodies (Sigma‐Aldrich). Signals were developed using the Clarity Western ECL Substrate (Bio‐Rad, Hercules, CA) and detected with a FUSION Solo S device (Vilber, France).

### 2.6 RNA isolation and qPCR

Total RNA was isolated using the GenElute Mammalian Total RNA Miniprep Kit (Sigma‐Aldrich, St. Louis, MO). cDNA was synthesized from 1 μg of total RNA using the QuantiTect Reverse Transcription Kit (Qiagen, Hilden). qPCR was performed using the KAPA SYBR FAST kit (Roche). Primer sequences are listed in Table S1.

### 2.7 Cell adhesion

Cells (1 × 10^3^) were seeded in 96‐well plates (coated with Matrigel or uncoated) and incubated for 1 h. Non‐adherent cells were washed away with sterile PBS. Attached cells were treated with 500 μg·mL^−1^ MTT for 1 h; formazan crystals were dissolved in DMSO, and absorbance was measured at 570 nm.

### 2.8 Cell proliferation

Proliferation of OSA cells under various conditions (complete medium, serum‐ and glutamine‐depleted medium, and hypoxia) was assessed using an MTT assay. Briefly, 2 × 10^3^ cells were seeded into 96‐well plates and incubated for 24–72 h. Cells were then treated with 500 μg·mL^−1^ MTT for 1 h, after which formazan crystals were dissolved in DMSO, and absorbance was measured at 570 nm.

### 2.9 Cell motility

Real‐time migration was analyzed using the xCELLigence DP instrument (Roche) with the RTCA CIM‐Plate 16 (Agilent, Santa Clara, CA), either coated with Matrigel or left uncoated. A total of 7 × 10^4^ pre‐starved cells (incubated in serum‐free medium for 3 h) were seeded, and migration was monitored every 15 min for 24 h.

*In vitro* wound closure was assessed using a scratch assay. Cells were plated and grown to confluence, starved overnight in serum‐free medium, and then scratched with a sterile 1000 μL pipette tip. Cells were further cultured in serum‐free medium, and photographs were taken at 0 h and 24 h for HOS cells, which migrate faster, and at 0 h, 24 h, and 48 h for SAOS‐2 cells. Images were processed using the imagej software, and the wound area was measured.

### 2.10 Spheroid formation and collagen migration assay

Spheroids from the HOS cell line (Mock, upCIN85, siNeg, and siCIN85) were generated according to the protocol described previously [24]. Cells were treated with siRNA 48 h before spheroid formation. After 3 days of cultivation in 3D conditions, spheroids were stained with CellTracker Red (C34552; ThermoFisher Scientific), embedded in collagen, imaged every 24 h for up to 72 h, and analyzed according to the protocol described in [25]. Number of Migrated Objects/Mask Perimeter parameter was used for the evaluation of spheroid invasion.

### 2.11 Gelatin zymography

Activity of MMP2 and MMP9 was evaluated using gelatin zymography. Conditioned medium was collected after 72 h of incubation. Gelatinolytic activity was assessed according to the Abcam protocol: https://www.abcam.com/protocols/gelatin‐zymography‐protocol.

### 2.12 RNA sequencing and functional enrichment analysis

RNA was isolated as described above. Quality control, library preparation, RNA sequencing, and differential expression analysis were performed by the Bioinformatics Core Facility, Centre for Molecular Medicine, CEITEC Masaryk University (Brno, Czech Republic). Briefly, RNA sequencing was carried out on an Illumina NextSeq 500 sequencer using 75‐bp single‐end reads, generating approximately 10 million reads per library. Mapped reads were counted and summarized at the gene level using featureCounts. Differential gene expression analysis was performed with the DESeq2 package. An adjusted *P*‐value of ≤ 0.05 and a fold‐change threshold of 1.5 were considered significant. Visualizations and downstream functional analyses were performed using R packages designed for bioinformatic workflows. For graphical representation of differential expression results, a volcano plot of the top 1000 DEGs ranked by adjusted *P*‐value was created using EnhancedVolcano. Normalized counts were used to generate a heatmap of the top 20 upregulated and downregulated genes.

Overrepresentation analysis of DEGs (using the GO Biological Process, KEGG, and MSigDB Hallmark databases) was performed with enrichR [26]. To summarize and reduce redundant GO terms, rrvgo was applied with a similarity threshold of 0.7 [27]. Additionally, Gene Set Enrichment Analysis (GSEA), based on the ranking metric sign(log_2_FoldChange) × (−log_10_(*P*‐value)), was performed using clusterProfiler. Venn diagrams of commonly enriched terms (*P* < 0.05 for the GO BP database and *P* < 0.25 for MSigDB and KEGG) were generated using the online tool available at the PSB Bioinformatics Webtools Venn page https://bioinformatics.psb.ugent.be/webtools/Venn/.

Protein–protein interactions were visualized with STRING (https://string‐db.org/), and hub genes were identified using the CytoHubba plugin in Cytoscape software based on their degree value. The CIN85 interactors list was obtained from the BioGRID database (https://thebiogrid.org/) [28].

### 2.13 Statistical analysis

Statistical analysis was performed using Origin software. Data are presented as mean ± SD unless otherwise specified in the figure legend, and individual data points for each replicate are shown. Normality of data distribution was assessed using the Shapiro–Wilk test. Comparisons between two groups were performed using a two‐tailed unpaired *t*‐test. For comparisons involving more than two groups, one‐way ANOVA followed by Tukey's multiple comparisons test was applied. The number of biological replicates is indicated in each figure legend, with a minimum of three replicates. Statistical significance was defined as: ns not significant; *P < 0.05; **P < 0.01; ***P < 0.001. Only statistically significant comparisons are shown unless stated otherwise.

## 3 Results

### 3.1 Expression of CIN85 in human OSA samples

**Fig. 1:**
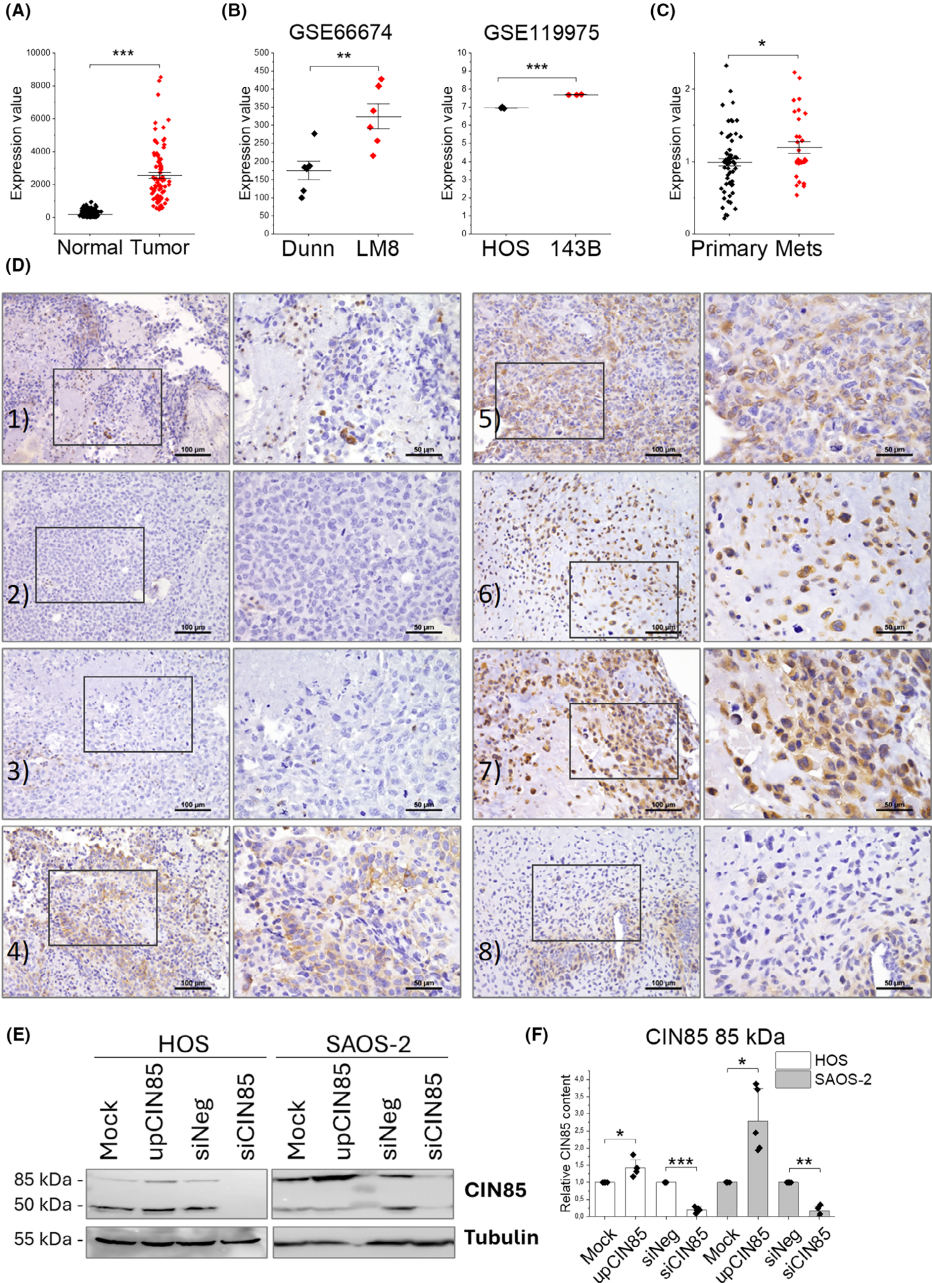
Datamining of CIN85 expression in osteosarcoma and characterization of generated cell sublines. (A) *SH3KBP1* mRNA expression in human osteosarcoma samples (OS‐TARGET dataset, *N* = 88) compared with normal bone; data retrieved from the TNMplot database; (B) *SH3KBP1* mRNA expression in human osteosarcoma cell lines (paired non‐metastatic and metastatic lines); data from GEO datasets (*n* = 6 for GSE66674; *n* = 3 for GSE119975); (C) *SH3KBP1* mRNA expression in human osteosarcoma primary tumors (Primary) versus metastases (Mets); data from GEO datasets (*N* = 96); (D) Immunohistochemical staining of CIN85 in human osteosarcoma tissue samples from the University Hospital Brno; representative images of 8 clinical samples shown at 200× and 400× magnification (scale bar 100 μm and 50 μm, respectively); (E) Representative western blot of CIN85 protein levels in generated osteosarcoma cell lines; (F) Densitometric quantification of CIN85 (85‐kDa isoform); tubulin served as a loading control (*n* = 4). Data are presented as mean ± SE (A–C) or mean ± SD (F), with individual data points shown. Statistical significance was assessed using a two‐tailed unpaired *t*‐test. For panels A‐C, F significance is indicated as follows: *P < 0.05; **P < 0.01; ***P < 0.001.

According to the TNMplot database, *SH3KBP1* expression is significantly higher in human OSA samples than in normal bone tissue (Fig. 1A). Analysis of publicly available datasets shows that *SH3KBP1* expression may vary between parental and highly metastatic OSA cell lines. Specifically, a significant difference was observed between the mouse Dunn (parental) and LM8 (metastatic) OSA cell lines, as well as between the human OSA cell lines HOS (parental) and 143B (metastatic) cultured on Matrigel, or the other metastatic cell lines KHOS and KRIB derived from HOS cells (Fig. 1B, Fig. S1A). Additionally, four datasets (GSE220538, GSE237033, GSE87624, and GSE32981) with patient samples were analyzed to compare *SH3KBP1* expression in OSA metastases versus primary tumors. Due to the relatively small number of patients in each dataset, no significant differences were detected individually. However, after normalizing and pooling data from all four datasets, *SH3KBP1* expression was significantly higher in metastases (Student's *t*‐test with Welch's correction: *P* = 0.03138, Mann–Whitney: *P* = 0.03869, Fig. 1C, Fig. S1B). To evaluate CIN85 expression at the protein level, we performed IHC staining in a cohort of pediatric OSA patients from the University Hospital Brno (Fig. 1D). CIN85 expression exhibited considerable heterogeneity, ranging from completely negative to highly positive samples. No significant correlation was observed between CIN85 IHC positivity and the presence of metastasis, nor did overall survival correlate with CIN85 protein levels in this small cohort of OSA patients (Table S2, Fig. S1C). Notably, all OSA cases with chondroblastic histology (*n* = 3, Fig. 1D, panels 6–8) showed CIN85 positivity, whereas only 15% of cases (2 out of 13) with the most frequent osteoblastic subtype were CIN85‐positive (Table S2).

To investigate the effect of CIN85 on OSA cell properties, sublines with different CIN85 expression levels were generated. The full‐length 85 kDa CIN85 isoform was overexpressed in HOS and SAOS‐2 cells (upCIN85), with mock‐transfected cells serving as controls. siRNA‐mediated knockdowns (siCIN85) and corresponding controls (siNeg) were also established for both HOS and SAOS‐2 cells, resulting in silencing all observed CIN85 isoforms (including the one with molecular weight around 56 kDa). CIN85 expression in the OSA sublines is shown in Fig. 1E,F.

### 3.2 CIN85 increases the motility of OSA cells *in vitro*

The adaptor protein CIN85 is known to interact with components of several signaling pathways and may therefore influence cancer cells' behavior. The list of CIN85 binding partners was retrieved from the BioGRID database, and enrichment analysis of this protein set indicated that CIN85 is involved in the regulation of cell adhesion, migration, cell death, and differentiation (Fig. S2). Based on these findings, we examined the proliferation of OSA cells with different levels of CIN85 expression under various conditions, including complete culture medium, serum and glutamine deprivation, hypoxia, and 3D growth. No significant effect of CIN85 on cell proliferation was observed (Fig. S3).

**Fig. 2:**
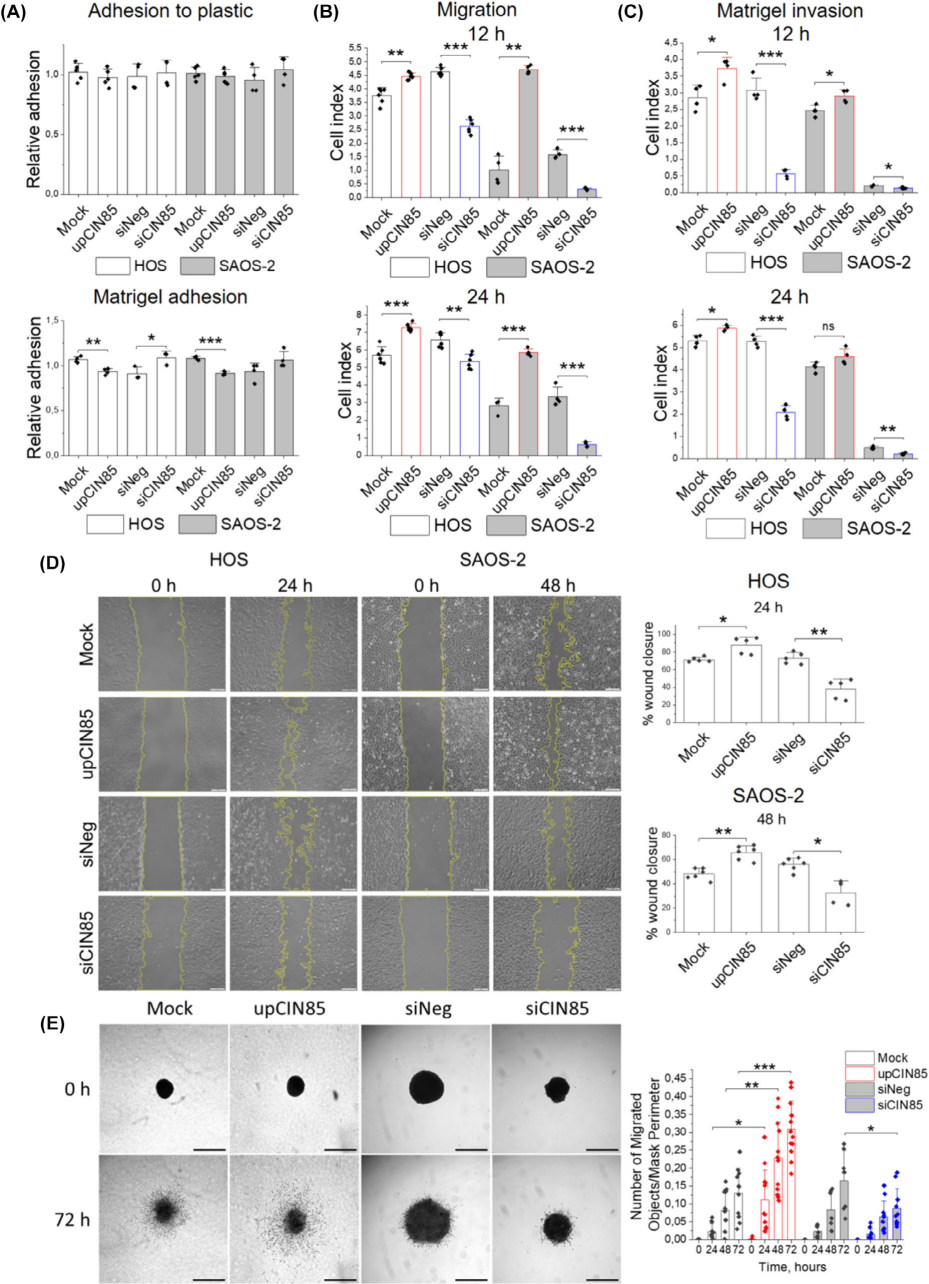
CIN85 affects osteosarcoma (OSA) cell adhesion and migration. (A) Adhesion to culture plastic and Matrigel, shown as values relative to the mock control of each indicated cell line; (B) xCELLigence real‐time migration, cell index is a relative change in measured impedance to represent cell status, *n* = 3; (C) xCELLigence real‐time Matrigel invasion, *n* = 3; (D) cell migration assessed by an *in vitro* scratch assay, images acquired at 40× magnification (scale bar: 200 μm); (E) 3D migration of collagen‐embedded HOS cell spheroids, images acquired at 50× magnification (scale bar: 500 μm). Data are presented as mean ± SD with individual data points shown. Sample sizes (*n*) were as follows: A upper panel—Mock, upCIN85: 5, siNeg, siCIN85: 4; (A) lower panel – HOS siNeg, siCIN85: 3, other groups: 4; (B) HOS cells: 6, SAOS‐2 cells: 4; (C): 4; (D) HOS cells: 5, SAOS‐2 Mock, upCIN85, siNeg: 6, SAOS‐2 siCIN85: 4; (E) Mock, upCIN85, siCIN85: 10, siNeg: 7. Statistical significance was assessed using a two‐tailed unpaired *t*‐test. Significance (panels A‐E) was defined as: ns ‐ not significant; *P < 0.05; **P < 0.01; ***P < 0.001. For panels A, E only statistically significant comparisons are shown.

Next, we assessed the adhesion of OSA cells to culture plastic and Matrigel in relation to CIN85 expression levels. A negative correlation between CIN85 expression and adhesion to Matrigel (but not to culture plastic) was observed (Fig. 2A). Real‐time motility was analyzed using the xCELLigence migration assay (Fig. 2B,C). High CIN85 expression was associated with increased migration (Fig. 2B) and Matrigel invasion (Fig. 2C), whereas CIN85 silencing resulted in a substantial reduction in both migration and invasion. Cell motility was further evaluated using an *in vitro* scratch assay, which demonstrated a significant increase in motility of CIN85‐overexpressing OSA cells and a decrease in siRNA‐treated cells (Fig. 2D). Finally, a 3D collagen invasion assay was performed using HOS cells with different levels of CIN85 expression (since SAOS‐2 cells do not form spheroids). These results confirmed the findings from the monolayer assays, showing that CIN85 enhances OSA cell motility (Fig. 2E).

### 3.3 Analysis of CIN85‐mediated transcriptomic changes in OSA cells

To investigate the transcriptomic basis and signaling deregulation underlying the observed functional changes in OSA cell behavior, RNA‐seq analysis was performed on HOS upCIN85, HOS siCIN85, and their respective control cells.

Using an adjusted *P*‐value threshold of < 0.05, 301 genes were differentially expressed between upCIN85 and Mock cells, including 107 upregulated and 194 downregulated transcripts. Applying an additional log_2_FC cutoff of 0.59 (corresponding to > 50% change) reduced this set to 172 DEGs, comprising 67 upregulated and 105 downregulated genes.

In the siCIN85 versus siNeg comparison, 298 genes met the adjusted *P*‐value criterion, with 134 upregulated and 164 downregulated. After applying the same log_2_FC cutoff, 143 DEGs remained, including 74 upregulated and 69 downregulated genes.

**Fig. 3:**
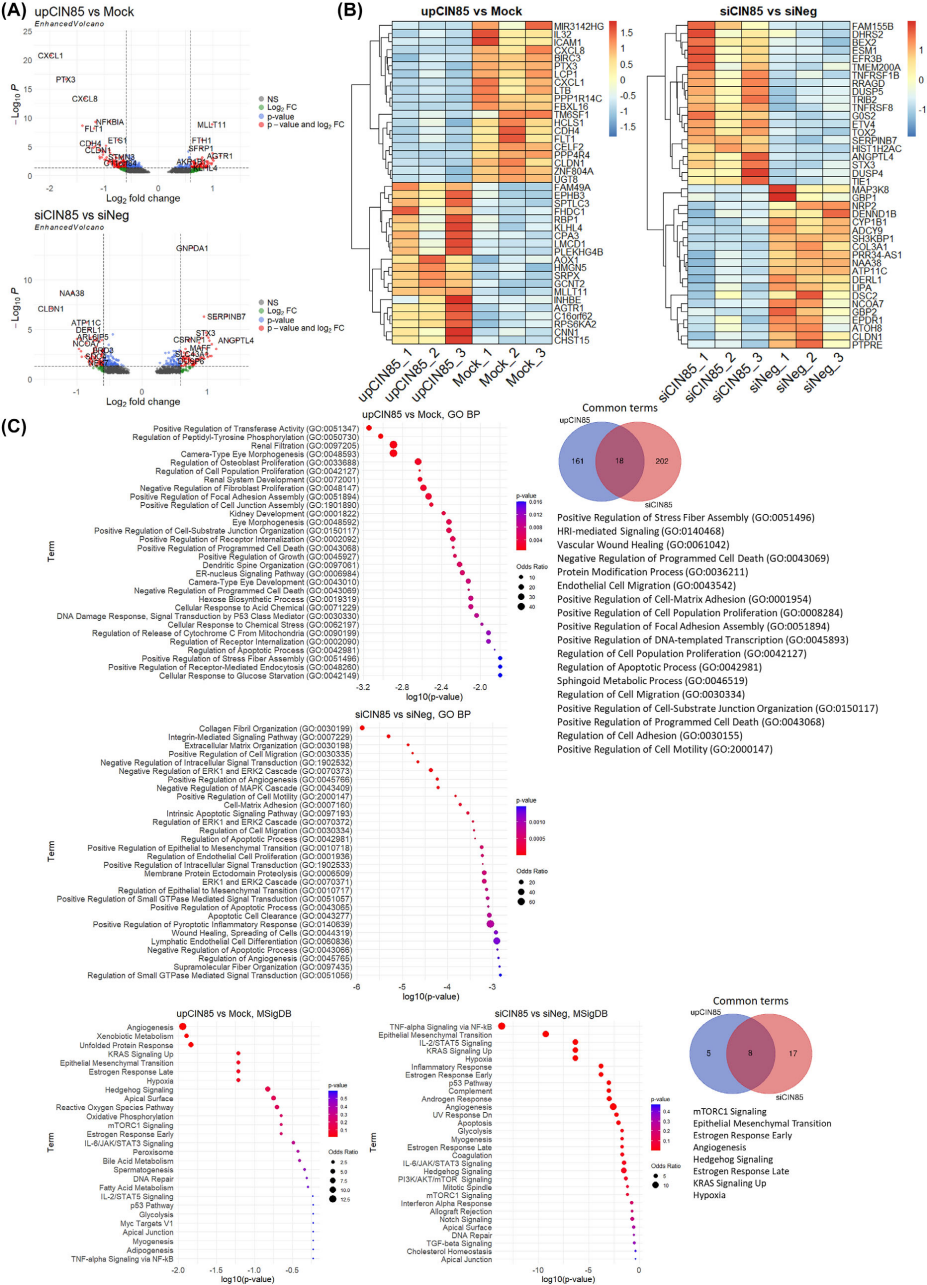
Transcriptomic analysis of HOS cells depending on CIN85 expression. (A) Differentially expressed genes (DEGs) visualized as a volcano plot. The *P*‐values shown in the volcano plot originate from the DESeq2 differential expression analysis, which uses a Wald test within a negative binomial generalized linear model. Color coding: red—significant by both adjusted *P*‐value and log_2_FC (0.05 and 0.59, respectively); blue—significant by adjusted *P*‐value only (0.05); green—significant by log_2_FC only (0.59); gray – not significant (NS). (B) Heatmap of the top 20 upregulated and top 20 downregulated genes, (C) Overrepresentation analysis of DEGs using GO Biological Process (GO BP) and Molecular Signatures Database (MSigDB) annotations; *P*‐values correspond to Fisher's exact test as implemented in enrichR; Venn diagrams show enriched terms shared between upCIN85 and siCIN85 conditions.

The top 1000 genes ranked by adjusted *P*‐value are shown in the volcano plot (Fig. 3A). Heatmaps displaying the top 20 upregulated and top 20 downregulated genes for the upCIN85 vs. mock and siCIN85 vs. siNeg comparisons are presented in Fig. 3B.

Eight genes were inversely regulated in both cell models—downregulated in upCIN85 cells and upregulated in siCIN85 cells: *HCLS1*, *PLPP2*, *ETS1*, *ESM1*, *DHRS2*, *ARHGDIB*, *ADAM19*, and *PHLDA2*. No common genes were identified among DEGs upregulated in upCIN85 and downregulated in siCIN85 cells. All genes deregulated by CIN85 (up‐ and downregulated in both conditions) were visualized in STRING as a protein–protein interaction network and analyzed in Cytoscape to identify the top 20 hub genes (Fig. S4).

To identify relevant biological processes and signaling pathways deregulated by CIN85, overrepresentation analysis of the DEG lists (adjusted *P*‐value < 0.05 and absolute log_2_FC > 0.59) was performed against the GO Biological Process, MSigDB, and KEGG databases (Fig. 3C, Fig. S5). Common significantly overrepresented terms in upCIN85 and siCIN85 cells included *Negative Regulation of Programmed Cell Death*, *Positive Regulation of Cell–Matrix Adhesion*, *Positive Regulation of Focal Adhesion Assembly*, *Regulation of Cell Population Proliferation*, *Regulation of Cell Migration*, *Regulation of Cell Adhesion*, *Positive Regulation of Cell Motility* (GO BP), *Epithelial–Mesenchymal Transition, mTORC1 signaling, Hypoxia* (MSigDB), as well as Wnt, MAPK, PI3K–Akt, and TNF signaling pathways (KEGG).

To summarize and reduce redundant GO Biological Process terms, the REVIGO algorithm was applied (Fig. S6). For the upCIN85 DEG list, identified clusters included regulation of translation in response to endoplasmic reticulum stress, regulation of cell population proliferation, positive regulation of cell junction assembly, skeletal muscle tissue regeneration, cellular response to oxidative stress, regulation of cell adhesion, regulation of cell migration, negative regulation of bone remodeling, and skeletal muscle fiber development. For the siCIN85 DEG list, clusters included collagen fibril organization, integrin‐mediated signaling pathway, positive regulation of cell migration, negative regulation of intracellular signal transduction, cell–matrix adhesion, intrinsic apoptotic signaling pathway, regulation of the ERK1 and ERK2 cascade, positive regulation of epithelial–mesenchymal transition, regulation of biomineral tissue development, and negative regulation of the G1/S transition of the mitotic cell cycle.

In addition, Gene Set Enrichment Analysis (GSEA) was performed to identify upregulated and downregulated biological processes and pathways (Figs S7–S9) In upCIN85 cells, enriched upregulated terms included, among others, *mTORC1 signaling* (MSigDB), whereas downregulated terms included *Positive Regulation of Extracellular Matrix Organization* and *Regulation of Homotypic Cell–Cell Adhesion*. In siCIN85 cells, upregulated terms included *TNFα Signaling* via *NF‐κB*, *Hypoxia*, and *IL2 STAT5 Signaling* (MSigDB). Downregulated terms included *Wound Healing*, *Extracellular Matrix Organization*, *Actin Filament–Based Movement*, and *Regulation of Cell–Substrate Adhesion* (GO BP).

Taken together, common enriched terms identified in DEGs of both upCIN85 and siCIN85 cells indicated that CIN85 is associated with proliferation, survival, migration, and adhesion in OSA cells and suggested potential signaling pathways involved, including MAPK, mTOR, PI3K/Akt, and NF‐κB.

### 3.4 Validation of transcriptomic analysis and investigation of CIN85‐driven mechanisms in OSA cells

Expression of several CIN85‐regulated genes (based on the RNA‐seq DEGs overview presented at Table S3) was further evaluated by qPCR in HOS and SAOS‐2 cells (Fig. S10). Specifically, qPCR confirmed significant changes in the expression of *ADAM19, DLX2, ARHGDIB*, and *HCLS1* in both HOS and SAOS‐2 cells under either overexpression or knockdown conditions. Other genes showed significant changes in only one cell line or in neither of them.

**Fig. 4:**
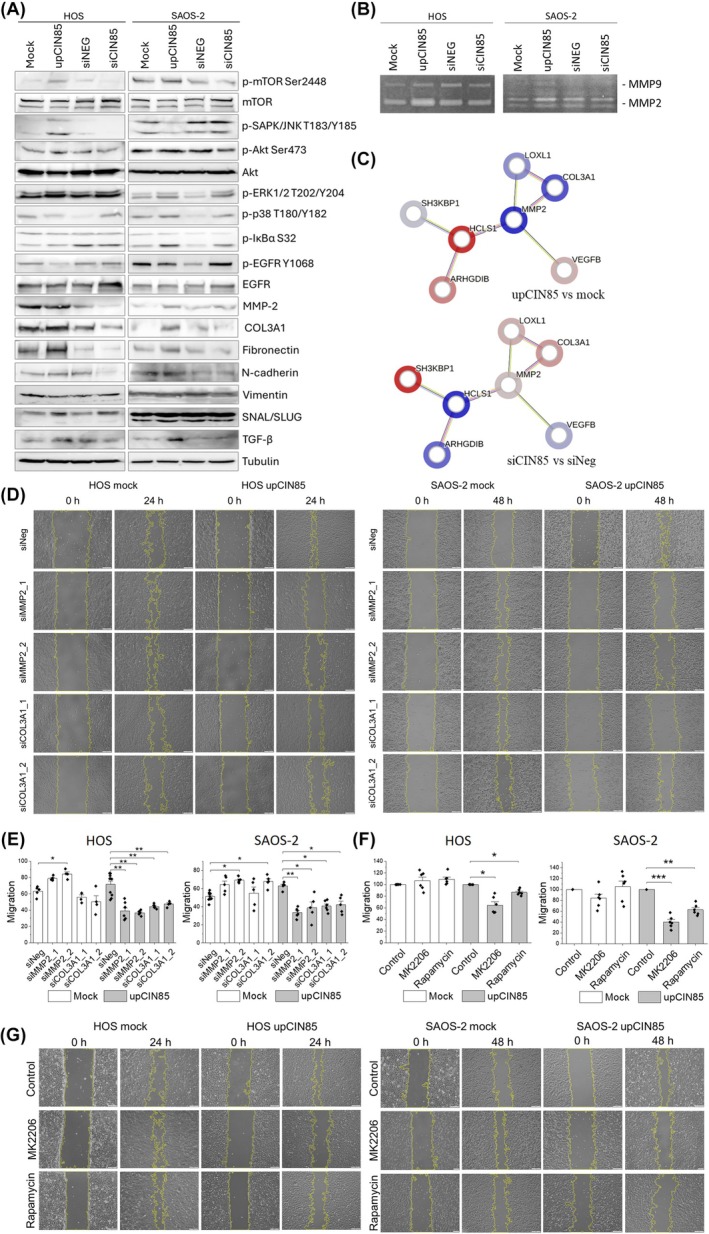
Analysis of molecular markers and validation of molecular mechanisms underlying the enhanced migration of CIN85‐overexpressing osteosarcoma (OSA) cells. (A) Representative western blot (*n* = 3), α‐tubulin was used as a loading control; (B) analysis of MMP2 and MMP9 activity by gelatin zymography (representative image, *n* = 3); (C) functional protein interaction network of migration‐related DEGs, including *SH3KBP1*, generated using the STRING database (interaction score = 0.4), red indicates decreased expression and blue indicates increased expression; (D, E) effect of *MMP2* and *COL3A1* silencing on OSA cell migration assessed by an *in vitro* scratch assay, images acquired at 40× magnification (scale bar: 200 μm); (F, G) effect of the inhibitors of mTOR (Rapamycin) and Akt (MK2206) signaling on OSA cell migration assessed by an *in vitro* scratch assay, images acquired at 40× magnification (scale bar: 200 μm). Data are presented as mean ± SD with individual data points shown. Sample sizes (*n*) were as follows: E – HOS Mock siNeg, siMMP2_1: 5; HOS Mock siMMP2_2, siCOL3A1_1, siCOL3A1_2: 4, HOS upCIN85 siNeg, siMMP2_1: 8; siMMP2_1, siCOL3A1_1: 6, siCOL3A1_2: 4; F: 6. Statistical significance was assessed using one‐way ANOVA followed by Tukey's multiple comparisons test. For panels E, F significance is indicated as follows: *P < 0.05; **P < 0.01; ***P < 0.001; only statistically significant comparisons are shown.

Western blotting was used to assess signaling pathway activation by analyzing phosphorylated proteins (Fig. 4A). CIN85 enhanced mTOR and Akt activation (Fig. 4A, Fig. S11), as well as TGFβ and fibronectin expression in both HOS and SAOS‐2 cells, while effects on SAPK/JNK, EGFR, ERK1/2, p38, and NF‐κB varied by cell line and condition. Western blotting and gelatin zymography also confirmed CIN85‐dependent changes in MMP2 and COL3A1 expression/activity (Fig. 4A,B).

To further investigate the functional relevance of CIN85‐regulated genes, we visualized their interactions using STRING. This analysis revealed a network of DEGs with opposing expression changes in upCIN85 and siCIN85 cells (Fig. 4C). *HCLS1, MMP2*, and *COL3A1* emerged as central nodes, while additional genes, including *ARHGDIB, LOXL1, and VEGFB*, also contributed to the network; all are known to play roles in cancer migration and metastasis [29, 30, 31, 32]. Survival analyses (Fig. S12) further indicated that high expression of *COL3A1* and *MMP2* is associated with shorter metastasis‐free survival in OSA patients, whereas high *HCLS1* expression predicts better prognosis. These results suggest that CIN85 may influence OSA progression through coordinated regulation of key metastasis‐related genes.

To assess the roles of *HCLS1, COL3A1*, and *MMP2* in OSA cell motility, we performed siRNA‐mediated knockdowns. Silencing *HCLS1* in parental cells had no significant effect on motility in either HOS or SAOS‐2 cells (Fig. S13). In contrast, *MMP2* knockdown in upCIN85 cells resulted in approximately 45% reduction in motility in both cell lines. Similarly, silencing *COL3A1* decreased motility by about 35% in HOS and SAOS‐2 upCIN85 cells. Notably, knockdown of these genes in mock‐transfected cells had little to no effect on motility, and in some cases slightly increased it (Fig. 4D,E, Fig. S14).

Gene enrichment analysis of CIN85‐regulated genes predicted involvement of the Akt/mTOR signaling pathway, which was confirmed by changes in p‐Akt and p‐mTOR levels (Fig. 4A, Fig. S11). To assess the functional impact of these pathways on cell motility, we treated cells with the Akt inhibitor MK‐2206 and the mTOR inhibitor rapamycin (Fig. 4F,G). CIN85‐overexpressing cells were more sensitive to pathway inhibition. Specifically, Akt inhibition reduced motility by approximately 30% in HOS upCIN85 cells and 50% in SAOS‐2 upCIN85 cells. mTOR inhibition also significantly suppressed motility, by 10% in HOS and 35% in SAOS‐2 CIN85‐overexpressing cells. In contrast, mock‐transfected cells showed no significant change in motility upon treatment with either inhibitor.

In summary, combined transcriptomic and functional analyses of HOS cells with altered CIN85 expression indicate that CIN85 promotes OSA cell motility by upregulation of *MMP2* and *COL3A1* and activation of the Akt/mTOR signaling pathway. These findings link CIN85‐dependent transcriptional changes to an increase in cell migration and invasion, highlighting potential molecular mechanisms driving OSA progression.

## 4 Discussion

Our study identifies the adaptor protein CIN85 as a key enhancer of osteosarcoma cell migration and collagen invasion. Increased CIN85 expression has been observed in various cancers compared to normal tissue, and high CIN85 expression is associated with poorer prognosis [10, 11, 12, 13, 14, 15, 16]. Here, we demonstrated a similar trend in osteosarcoma: CIN85 expression is elevated in OSA tumors compared to normal bone and is higher in metastases than in primary tumors. Notably, all analyzed samples of the chondroblastic subtype of OSA—characterized by poor prognosis, high recurrence rates, and pronounced metastatic potential [33, 34]—were CIN85‐positive (Table S2), underscoring the possible relevance of CIN85 expression in disease progression. Consistent with this, a transcriptomic study previously identified *SH3KBP1* as one of the genes overexpressed in metastatic OSA cell lines [18].

To investigate the functional involvement of CIN85 in OSA cells, we generated HOS and SAOS‐2 sublines with overexpression of the full‐length CIN85 (upCIN85) and CIN85 knockdown (siCIN85). CIN85 was shown to modulate cell adhesion and significantly enhance migration. These findings are consistent with previous studies demonstrating that CIN85 promotes migration, invasion, and metastasis in other cancer types, such as breast cancer [10, 11, 35], melanoma [10], and esophageal squamous cell carcinoma [14].

CIN85 participates in multiple oncogenic signaling pathways across diverse cancer types. It stabilizes HIF1α, enabling HIF‐dependent transcription even under normoxia [36, 37], and amplifies TGFβ‐induced EMT signaling [12], a key driver of metastatic progression [38, 39]. CIN85 also regulates Src‐dependent processes such as cell adhesion, invadopodia formation, and motility [9, 11, 40, 41]. As part of the CBL–CIN85–endophilin complex, CIN85 promotes endocytosis of EGFR [42] and other receptors [43, 44, 45], thereby regulating diverse signaling pathways [46]. It directly interacts with MEKK4, activating the MKK6/p38 MAPK cascade [47], and binds the PI3K regulatory subunit p85α, modulating PI3K/Akt signaling [48, 49]. CIN85 further influences cancer cell invasiveness through interactions with AIP1, MUC1, F‐actin, AMAP1, N‐WASP, and MT1‐MMP [10, 15, 17, 41, 50, 51]. Together, these interactions position CIN85 as a multifunctional scaffold that integrates RTK trafficking, cytoskeletal remodeling, and pro‐metastatic signaling.

Functional enrichment analysis of RNA‐seq data from HOS cells with CIN85 overexpression or knockdown revealed significant overrepresentation of genes annotated to several cancer‐related signaling pathways, including TNF, NF‐κB, MAPK, TGFβ, and PI3K/Akt/mTOR. In this study, we demonstrated that CIN85 overexpression in OSA cells potentiates the Akt/mTOR signaling, while pharmacological inhibition of these pathways reduces the migration rate of CIN85‐overexpressing cells. Recent studies have shown that inhibition of Akt/mTOR signaling suppresses osteosarcoma tumor growth *in vivo* [52, 53], underscoring the critical role of this pathway in OSA progression. Furthermore, the PI3K/Akt/mTOR axis is implicated in metabolic reprogramming and the Warburg effect in OSA cells, thereby promoting invasion and metastasis [2]. Everolimus, a rapamycin‐derived mTORC1 inhibitor, uniquely combines antitumor and osteoprotective effects by suppressing metabolic reprogramming, angiogenesis, and osteoclastogenesis. Refining patient selection through molecular profiling remains a key priority to establish everolimus as a precision therapeutic platform for OSA [8]. Our results indicate that CIN85‐driven OSA may respond more favorably to Akt/mTOR inhibition than CIN85‐null tumors.

We also identified *MMP2* and *COL3A1* as key genes involved in CIN85‐dependent migration of OSA cells. *MMP2* is one of the most extensively studied genes in cancer biology; it facilitates extracellular matrix degradation, regulates cell adhesion, and promotes migration and metastasis in various cancers, including OSA [54]. *COL3A1* was identified as one of the top 20 hub genes in the network of CIN85‐induced DEGs. Its overexpression in OSA cells has been associated with methotrexate resistance and regulation of apoptosis [55]. Although *MMP2* and *COL3A1* do not interact directly, they are linked via the adhesion molecules FN1, ITGB1, CD44, and THBS1 (Fig. S15). Another study demonstrated that both *MMP2* and *COL3A1*, along with *CDH2* and *TWIST1*, were downregulated in OSA cells upon knockdown of the invasion‐related protein LAMP3 [46]. Collectively, these findings suggest that CIN85 promotes OSA cell migration at least partially through the *MMP2‐COL3A1* axis. Importantly, previous evidence indicates that inhibition of PI3K/Akt/mTOR signaling reduces *MMP2* expression [56, 57, 58, 59], and there is also a reported link between *COL3A1* and PI3K/Akt signaling [60, 61].

Considering the accumulated evidence on the role of CIN85 in cancer migration and metastasis, this adaptor protein may represent a promising therapeutic target. Recent studies have explored CIN85 interactions with the glycoprotein MUC1, which is involved in cancer metastasis, and have identified possible druggable sites within the CIN85 molecule [62, 63]. Additionally, CIN85 interacts with PHD2, stabilizing HIF‐1α and inducing the expression of HIF‐dependent genes, such as *LDHA, GLUT1, CITED2*, and *VEGF*, all of which contribute to cancer progression [37]. Our results showed that CIN85‐driven migration depends on *MMP2*, *COL3A1*, and the Akt/mTOR pathway. Beyond targeting CIN85 itself, inhibiting downstream effectors identified in this study may also provide therapeutic benefit in CIN85‐driven tumors.

## 5 Conclusions

In summary, we demonstrated that the adaptor protein CIN85 is overexpressed in osteosarcoma and that its increased expression correlates with metastasis. Using OSA cell lines with CIN85 overexpression or knockdown, we showed that CIN85 enhances migration and invasion of OSA cells. Transcriptome profiling of HOS cells with altered CIN85 expression identified numerous differentially expressed genes and enriched signaling pathways. Mechanistically, increased migration of CIN85‐overexpressing OSA cells requires *MMP2* and *COL3A1* expression and activation of the Akt/mTOR signaling pathway. These findings highlight CIN85 as a central component of several dysregulated pathways, serving as a potential biomarker and therapeutic target in OSA, and warrant further clinical evaluation to assess its role in disease progression, metastasis, and a response to the targeted therapy.

## Conflict of interest

The authors declare no conflict of interest.

## Author contributions

IH and LK conceptualized the study, designed the experiments, and wrote the manuscript. IH and MP performed *in vitro* experiments; ISZ, PM, and DZ participated in clinical data collection and IHC. IH performed statistical analysis. JaN, MP, and JiN performed 3D spheroid growth and invasion experiments. LK, PB, JS, and LD supervised the project. All authors reviewed and approved the manuscript and contributed to the manuscript preparation.

## Supporting information


**Fig. S1.** *SH3KBP1* expression in human osteosarcoma.
**Fig. S2.** Enrichment analysis of CIN85 binding partners illustrating its cellular functions.
**Fig. S3.** CIN85 has little or no effect on osteosarcoma cell proliferation.
**Fig. S4.** Top 20 hub genes among CIN85‐regulated DEGs identified with Cytoscape.
**Fig. S5.** Overrepresentation analysis of CIN85‐regulated DEGs using the KEGG (Kyoto Encyclopedia of Genes and Genomes) database.
**Fig. S6.** Summary and visualization of GO (Gene Ontology) overrepresentation analysis.
**Fig. S7.** GSEA (Gene Set Enrichment Analysis) of CIN85‐regulated DEGs using the GO Biological Process database.
**Fig. S8.** GSEA (Gene Set Enrichment Analysis) of CIN85‐regulated DEGs using the MSigDB database.
**Fig. S9.** GSEA (Gene Set Enrichment Analysis) of CIN85‐regulated DEGs using the KEGG database.
**Fig. S10.** Validation of the expression of selected genes using qPCR.
**Fig. S11.** Densitometric analysis of phosphorylated Akt and mTOR from immunoblots.
**Fig. S12.** Survival plots of osteosarcoma patients based on the expression of *HCLS1*, *COL3A1*, and *MMP2*.
**Fig. S13.** The effect of *HCLS1* silencing on osteosarcoma cell migration.
**Fig. S14.** Immunoblot analysis of MMP2 (A) and COL3A1 (B) following siRNA‐mediated silencing.
**Fig. S15.** *MMP2* and *COL3A1* are linked through adhesion‐related molecules.
**Table S1.** List of primers used for qPCR.
**Table S2.** Clinicopathological features of osteosarcoma patients from the University Hospital Brno.
**Table S3.** Overview of CIN85‐regulated DEGs.

## Data Availability

Transcriptomics data generated during this study are openly available in the GEO DataSets database under the access number GSE292011.
