# Supplementary material for: Adaptor protein CIN85 potentiates the motility of osteosarcoma cells via the Akt/mTOR and MMP2‐COL3A1 axis
Source: Mol Oncol. 2026 Apr 17;20(8):2085–101. doi: 10.1002/1878-0261.70245 (PMC13398906; doi:10.1002/1878-0261.70245)
Supplement: Supplementary file 1 [file MOL2-20-2085-s001.docx]

**Supplementary Information**

***Horak I et al***

**Adaptor protein CIN85 potentiates the motility of osteosarcoma cells via the Akt/mTOR and MMP2-COL3A1 axis**

Fig. S1. *SH3KBP1* expression in human osteosarcoma. A - *SH3KBP1* mRNA expression in human osteosarcoma cell lines (HOS – non/low-metastatic, KHOS and KRIB – metastatic), n=3; B - *SH3KBP1* mRNA expression in human osteosarcoma primary tumors (Primary) or metastases (Mets) in each GEO dataset separately (GSE220538: N=33, GSE237033: N=17, GSE87624: N=25, and GSE32981: N=22); C - Survival plot of osteosarcoma patients from the University Hospital Brno stratified by CIN85 expression, N=20.

Data are presented as mean ± SE (A, B) with individual data points shown. Statistical significance was assessed using a two‑tailed unpaired t‑test. Significance is indicated as follows: *p < 0.05; **p < 0.01; ***p < 0.001.

Fig. S2. Enrichment analysis of CIN85 binding partners illustrating its cellular functions. A list of 329 CIN85 interacting proteins was obtained from the BioGRID database and subjected to overrepresentation analysis using the Gene Ontology (GO) annotations. The size of each enriched GO term reflects the number of proteins associated with that functional category.

Fig. S3. CIN85 has little or no effect on osteosarcoma cell proliferation. Proliferation of HOS and SAOS‑2 cells with different levels of CIN85 expression was assessed under standard culture conditions (complete medium), serum or glutamine deprivation (0.1% FBS or glutamine free medium), and hypoxia (1% O₂) using the MTT assay (n=3). To evaluate the effect of CIN85 on cell growth in 3D conditions, the growth of collagen‑embedded spheroids was monitored in HOS cells only (n=6), as SAOS‑2 cells are unable to form spheroids.

Data are presented as mean ± SD. Statistical significance was assessed using a two‑tailed unpaired t-test.

Fig. S4. Top 20 hub genes among CIN85-regulated DEGs identified with Cytoscape. All genes deregulated by CIN85 (both up‑ and downregulated under both experimental conditions) were visualized in STRING as a protein–protein interaction network and analyzed in Cytoscape to identify the top 20 hub genes. These hub genes formed three major clusters corresponding to: (I) collagen fibril organization and cell–matrix adhesion, (II) chemokine‑mediated signaling, and (III) cell‑cycle progression.

Fig. S5. Overrepresentation analysis of CIN85-regulated DEGs using the KEGG (Kyoto Encyclopedia of Genes and Genomes) database. The list of significant DEGs (as defined in Results 3.3 and shown in Fig. 3C) was subjected to overrepresentation analysis using the KEGG database. The top 30 enriched pathways, ranked by p-value, are displayed. p‑values correspond to Fisher’s exact test as implemented in enrichR. The Venn diagram illustrates KEGG terms commonly enriched in both upCIN85 and siCIN85 conditions.

Fig. S6. Summary and visualization of GO (Gene Ontology) overrepresentation analysis. The list of overrepresented Gene Ontology (GO) terms derived from CIN85 regulated DEGs was analyzed using the REVIGO algorithm to reduce redundancy and visualize major functional categories.

Fig. S7. GSEA (Gene Set Enrichment Analysis) of CIN85-regulated DEGs using the GO Biological Process database. Analysis was performed on the ranked list of genes identified by RNA‑seq using the GO Biological Process database to identify pathways enriched upon CIN85 modulation. Panels display normalized enrichment scores (NES) and gene counts for the top 50 enriched terms, ranked by p‑value. The p‑values shown correspond to the nominal p‑values generated by the GSEA permutation test.

Fig. S8. GSEA (Gene Set Enrichment Analysis) of CIN85 DEGs using the MSigDB database. Analysis was performed on the ranked list of genes identified by RNA‑seq using the MSigDB database to identify pathways enriched upon CIN85 modulation. Panels display normalized enrichment scores (NES) and gene counts for the top 50 enriched terms, ranked by p‑value. The p‑values shown correspond to the nominal p‑values generated by the GSEA permutation test.

Fig. S9. GSEA (Gene Set Enrichment Analysis) of CIN85 DEGs using the KEGG database. Analysis was performed on the ranked list of genes identified by RNA‑seq using KEGG database to identify pathways enriched upon CIN85 modulation. Panels display normalized enrichment scores (NES) and gene counts for all enriched terms, ranked by p‑value. The p‑values shown correspond to the nominal p‑values generated by the GSEA permutation test.

Fig. S10. Validation of the expression of selected genes using qPCR. *GAPDH* was used as the reference gene, n=3. Values of 2^(-dCp)^ are presented as mean ± SD with individual data points shown. Statistical significance was assessed using a two‑tailed unpaired t-test. Significance is indicated as follows: * p < 0.05; ** p < 0.01; *** p < 0.001.

Fig. S11. Densitometric analysis of phosphorylated Akt and mTOR from immunoblots. Band intensities from Fig. 4A were quantified and normalized to total Akt or total mTOR, n = 3.

Data are presented as mean ± SD with individual data points shown. Statistical significance was assessed using a two tailed unpaired t-test. Significance is indicated as follows: *p < 0.05; **p < 0.01; ***p < 0.001.

Fig. S12. Survival plots of osteosarcoma patients based on the expression of *HCLS1*, *COL3A1*, and *MMP2*. Survival data were retrieved from the R2 Genomics Analysis and Visualization Platform. The Mixed Osteosarcoma dataset (GSE42352, N=88) was used for the analysis, and metastasis‑free survival was evaluated with all other parameters kept at their default settings.

Survival differences were assessed using the log‑rank test. HCLS1: p = 3.7e^-3^, Bonferroni‑corrected p = 0.270; MMP2: p = 0.129, Bonferroni‑corrected p = 1; COL3A1: p = 6.96e^-3^, Bonferroni‑corrected p = 0.508.

Fig. S13. Effect of *HCLS1* silencing on osteosarcoma cell migration. Cell migration was assessed using an *in vitro* scratch assay, images were acquired at 40× magnification (scale bar: 200 µm), n = 4.

Migration data (% wound closure compared to day 0) are presented as mean ± SD with individual data points shown. Statistical significance was evaluated using a two‑tailed unpaired t‑test, no statistically significant differences were observed.

Fig. S14. Immunoblot analysis of MMP2 (A) and COL3A1 (B) following siRNA mediated silencing. Protein levels were assessed by Western blotting, and band intensities were normalized to tubulin, n = 3.

Data are presented as mean ± SD with individual data points shown. Statistical significance was evaluated using one-way ANOVA followed by Tukey’s multiple comparisons test. Significance is indicated as follows: *p < 0.05; **p < 0.01; ***p < 0.001.

Fig. S15. *MMP2* and *COL3A1* are linked through adhesion‑related molecules. A functional interaction network generated using MMP2 and COL3A1 as input was visualized in STRING with an interaction score of 0.9 (highest confidence). The number of interactors in the second shell was limited to 50, and all other parameters were left at their default settings.

Table S1. List of primers used for qPCR.

| **Gene** | **Forward primer** | **Reverse primer** |
| --- | --- | --- |
| *ADAM19* | CTCTGCTTGCTGGCGTTT | ATAAGGTGTTGGCCCTTGCT |
| *DLX2* | TACACCTCCTACGCTCCCTA | GTACCAGGGGTAGTTGCCCA |
| *ARHGDIB* | AGAAAACGCTGCTGGGAGAT | TCCACTTTCACCCCAGTCCT |
| *DHRS2* | ATGAGCAGCACCGGGATAGA | TTCCCATGAAACCAATCCTCACC |
| *ESM1* | AAAGACCACGACTGGAGAGC | TCAGGCATTTTCCCGTCCC |
| *HCLS1* | TACTCTCGTGGCTTTGGTGG | GCTCCTCTTTGTCACAGCCT |
| *EPHA2* | ATTAAGGACTCGGGGCAGGAG | TGCATCAGGTCCCACCCTTT |
| *COL3A1* | CGGAAACACTGGTGGACAGA | GTAGTCTCACAGCCTTGCGT |
| *LOXL1* | CGACCAGGGTTTCGTGTACT | TGGGGAGGAAGTCTGCTGT |
| *MMP2* | ATTTGGCGGACTGTGACGC | CAGGGTGCTGGCTGAGTAGAT |
| *GAPDH* | TCGGAGTCAACGGATTTGGT | TTCCCGTTCTCAGCCTTGAC |

Table S2. Clinicopathological features of osteosarcoma patients from the University Hospital Brno.

|  |  | **number** | **CIN85+ OSA** | **% of CIN85+ cells** |
| --- | --- | --- | --- | --- |
| **gender** | male | 12 | 4/12 (33 %) | 21.67 (0 - 100) |
|  | female | 8 | 2/8 (25 %) | 11.25 (0 - 80) |
| **histology** | chondroblastic | 3 | 3/3 (100 %) | 66.67 (10 - 100) |
|  | fibroblastic | 1 | 0/1 (0 %) | 0 |
|  | osteoblastic | 13 | 2/13 (15 %) | 11.15 (0 - 80) |
|  | teleangiectatic | 1 | 1/1 (100 %) | 5 |
|  | other | 2 | 0/2 (0 %) | 0 |
| **localization** | femur | 6 | 2/6 (33 %) | 14.17 (0 - 80) |
|  | tibia | 1 | 0/1 (0 %) | 0 |
|  | others | 13 | 4/13 (31 %) | 20.38 (0 - 100) |
| **metastasis** | no | 14 | 4/14 (29 %) | 18.93 (0 - 100) |
|  | yes | 6 | 2/6 (33 %) | 14.17 (0 - 80) |

Table S3. Overview of CIN85-regulated DEGs.

Identified CIN85 DEGs were analyzed to determine common genes among those up‑ or downregulated under upCIN85 or siCIN85 conditions. These DEGs were compared to each other (I), to the list of CIN85 binding partners (II and III), to genes correlating with *SH3KBP1* in osteosarcoma (OSA) samples (IV), to OSA metastasis (V), and to metastatic OSA cell lines (VI–IX).

| **upCIN85 vs Mock** | | **siCIN85 vs siNeg** | |
| --- | --- | --- | --- |
| *p<0.05 and logFC >0* | *p<0.05 and logFC <0* | *p<0.05 and logFC >0* | *p<0.05 and logFC <0* |
| **I. Common DEGs** | | | |
|  | *PLPP2, ADAM19, ETS1, PHLDA2, HCLS1, DHRS2, ESM1, ARHGDIB* | *PLPP2, ADAM19, ETS1, PHLDA2, HCLS1, DHRS2, ESM1, ARHGDIB* |  |
| **II. Binding patrners (BioGRID)** | | | |
| *NELFA, PPP3CA, ACTC1* | *PHLDB1, CBL, ARAP1, BIRC2* |  | *PPP3CB, ANLN, PDGFRB, SH3KBP1, DAG1, PLOD2, PHLDB2, ERC1* |
| **III. Binding patrners (STRING)** | | | |
|  | *CBL, ARAP1, PICALM* |  | *ANLN, SH3KBP1* |
| **IV. Correlation (TNMplot)** | | | |
| **r >0.3** | **r<0.3** | **r<0.3** | **r >0.3** |
| *MMP2, SLC16A2, EPHB3, FHAD1, CDH3, TRIM38, SRPX* | *DHRS2, MCAM, ID3, DLX2, SLC17A9, EFNA1, NOL4L, SELENOS, TMEM132A, MTSS1* | *DHRS2, TIE1, GRAMD1B, YRDC, PREX1, CLTB* | *ADRA2A, PRRG1, OGFRL1, LAYN, VCL, SLFN5, ETV6, TGFBI, ARSJ, EPS8, LOXL1, PDGFRB, SH3KBP1, NNMT, COL3A1, TPM4, NUDT11, COL16A1, MORC4, PHLDB2* |
| **V. GSE21257 OSA Metastasis vs No metastasis** | | | |
| **upregulated in Metastasis** | **downregulated in Metastasis** | **downregulated in Metastasis** | **upregulated in Metastasis** |
|  | *HCLS1, OGFRL1, CTSS, TM6SF1* | *HCLS1, PEA15, TNFRSF1B* |  |
| **VI. GSE66673 143B vs HOS (Metastatic vs non-metastatic cell line)** | | | |
| **upregulated in 143B** | **downregulated in 143B** | **downregulated in 143B** | **upregulated in 143B** |
| *SPP1, SLC16A2* | *ANXA3, NPPB, BDKRB1, COL18A1, ADRA2A, OLFM1, NIPAL4, EFNA1, OLR1, NAV2, BGN, TAGLN, SHE, INA, IL32, NMU, CLDN1, SLC27A2* | *BEX2, TNFRSF8* |  |
| **VII. GSE66673 LM5 vs SAOS-2 (Metastatic vs non-metastatic cell line)** | | | |
| **upregulated in LM5** | **downregulated in LM5** | **downregulated in LM5** | **upregulated in LM5** |
| *SERPINF1, ZNF721, SPP1, RAB15, S100A16, ACSS1, NKD2, CPE, TMEM2* | *FOXC2, SHISA3, LCP1, NAV2, DRAM1, IGFBP2, TGFBI* | *SERPINB7, TOX2, EFR3B* | *PDGFRB, AGA, RBL1* |
| **VIII. GSE66675 Dunn vs LM8 (Metastatic vs non-metastatic cell line)** | | | |
| **upregulated in LM8** | **downregulated in LM8** | **downregulated in LM8** | **upregulated in LM8** |
| *MMP2, CRNKL1, GYPC, PSMG4, LMO4, IDS, FHDC1, VEGFA, NSFL1C, ESYT2, NDUFB2, CPE, COL1A2, PSPH, FTL, MRFAP1L1, MRPS26, NUDT3, ACSS1, NKD2, RAB12, INSR, RBM8A, CNN1, PBX1, FTH1, FHAD1, S100A16, GATA3, RPS2, SOX12, CTBP1, AGTR1, SLC6A9, SLC4A11* | *ETS1, IRAK2, NFE2L3, ADRA2A, PRRG1, LCP1, ITGB3, USP53, CCL2* | *ETS1, DUSP5, NRIP3, SFMBT2, G0S2* | *SAMD4A, CDC25B, COL1A1, DDHD2, SDC2, UQCRQ, HINT1, EPDR1, SCOC, CTDSPL2, KATNAL1, MAP3K8, RRAGA, UBA3, MCAM, COL3A1, DENND1B, TDG, IQGAP1, SGMS2, DUT, ARL3, VANGL1, MCM6, ADCY9, RBL1, POLR2G, ZCRB1, ANLN, SPPL3, COL5A2, AGA, PPP3CB, ATF2, COL16A1, DLGAP5, P4HA1, CDKN2C, LRRC57, ANXA7, CALM1, PHLDB2, ANXA1, MGST3, NRP2, EVI5, LITAF, NEK7, USP13, SDC4, ITGAV, PCYOX1, PRMT6, BFAR, SCARB2, GOLIM4, MORC4, GMNN* |
| **IX. GSE49003 Metastatic vs non-metastatic cell line** | | | |
| **upregulated in Metastasis** | **downregulated in Metastasis** | **downregulated in Metastasis** | **upregulated in Metastasis** |
| *GYPC, MOK, POLR3GL, S100A16, SLC4A11, SFRP1, ESYT2* | *TAGLN, ID3, LAYN, PDLIM7, DUSP1, OLR1, MAOA, NFKBIA, CCNA2* |  | *SH3KBP1* |
